# Molecular survey of duck circovirus infection in poultry in southern and southwestern China during 2018 and 2019

**DOI:** 10.1186/s12917-020-02301-x

**Published:** 2020-03-05

**Authors:** Hao Liu, Li Xia Li, Wen Chao Sun, Ning Shi, Xiu Tao Sun, Ning Yi Jin, Xing Kui Si

**Affiliations:** 1 School of Life Sciences and Engineering, Foshan University, Foshan, 528000 China; 2 Jilin Wildlife Rescue and Rehabilitation Center, Forestry Department of Jilin Province, Changchun, 130122 China; 3 Institute of Virology, Wenzhou University, Wenzhou, 325035 China; 4 Honghe Animal Disease Prevention and Control Center, Mengzi, 661000 China; 5 Military Veterinary Institute, Academy of Military Medical Sciences, Changchun, 130122 China

**Keywords:** Duck circovirus, Epidemiological investigation, Phylogeny

## Abstract

**Background:**

Duck circovirus (DuCV) is a potential immunosuppressive virus that causes feather disorders in young ducks. In this study, DuCV obtained from various species of ducks was investigated by polymerase chain reaction (PCR) in southern and southwestern China (Guangdong, Guangxi and Yunnan provinces) from 2018 to 2019.

**Results:**

A total of 848 bursa samples were collected from dead Mulard, Cherry Valley Pekin, Muscovy and Mallard ducks from duck farms. The positivity rate of DuCV in the total sample was approximately 36.91%. We found that the prevalence of DuCV in Yunnan (43.09%) was higher than those in Guangxi (34.38%) and Guangdong (34.4%). However, the positivity rates of DuCV in the four duck species were not significantly different (*P* > 0.05). Nineteen randomly selected complete viral genomes were sequenced. The complete genomes of the DuCV were 1987 to 1995 nt in length, and were 81.7–99.3% homologous to the other 57 sequences in GenBank. Phylogenetic analyses based on the complete genomes of 76 DuCVs showed that the 19 novel DuCV sequences from Guangdong and Guangxi provinces mainly belonged to the DuCV-1 and DuCV-2 genetic groups, respectively. However, the two genotype groups coexisted in Yunnan Province. In addition, recombination analysis showed putative recombination sites in 3 strains in Yunnan that originated from strains Guangdong and Guangxi. Interestingly, the epidemiological investigation showed that Mulard ducks, Cherry Valley Pekin ducks and Muscovy ducks more than 4 weeks old were more susceptible to infection with the novel DuCV than ducks less than 4 weeks old.

**Conclusions:**

These data provide insight into the molecular epidemiology and genetic diversity of DuCVs circulating in southern and southwestern China for the first time.

## Background

The circovirus family (Circovirus) is a small, round, nonenveloped, single-stranded DNA virus with a circular genome of less than 2 kb [1, 2]. The Circovirus genus genome organization is ambisense, meaning that the two main open reading frames (ORFs) are responsible for encoding replication proteins (Rep) and capsid proteins (Cap). Members include porcine circovirus type 1 (PCV1), porcine circovirus type 2 (PCV2), porcine circovirus type 3 (PCV3), duck circovirus (DuCV), goose circovirus (GoCV), canary circovirus (CaCV), gull circovirus (GuCV) and other circoviruses [3–9].

DuCV was first reported in Germany in 2003 [5, 10]. Since then, DuCV has been reported in a number of countries, including Hungary, China, the US, Italy and South Korea [11–18]. DuCV mainly invades the host’s immune system and causes multiple secondary infections in domestic ducks [5, 6, 10, 17]. It can cause a variety of clinical symptoms, such as feathering disorders, growth retardation, low body weight (BW), lymphocyte depletion, necrosis, and histiocytosis in the bursa of Fabricius (BF) [10]. DuCV can be divided into two genotypes, DuCV-1 and DuCV-2, based on the complete genome and cap gene [17, 19]. Worldwide, there are more reports of DuCV-1 in Germany, Hungary, the US, China, South Korea, and Poland than DuCV-2 reports in Taiwan and China [17]. Recent research has suggested that DuCVs-1 and 2 are mainly concentrated in eastern coastal cities and in migrating wild ducks in China [20]. Epidemics of DuCVs-1 and 2 have been associated with increasingly serious harm to the poultry industry in northern and southern China, including Hebei, Shandong, Fujian and Zhejiang provinces [15, 17, 21]. However, there have been few reports of DuCV epidemic outbreaks in Guangdong, Guangxi and Yunnan provinces. Therefore, this research aimed to better understand the epidemiological and genetic characteristics of DuCV in southern and southwestern China in 2018 and 2019.

## Results

### Molecular assays of clinical samples

In this study, a total of 313 (36.91%) ducks were positive for DuCV. The positivity rates of DuCV were 34.4% (86/250), 34.38% (121/352) and 43.09% (106/246) in Guangdong, Guangxi and Yunnan provinces, respectively (Table 1). Among the positive samples, 39.47, 31.75 and 43.68% of Mulard ducks were positive; 30.0, 41.77 and 51.58% of Cherry Valley Pekin ducks were positive; 40.74, 30.61 and 39.76% of Muscovy ducks were positive; and 20.69, 33.67 and 42.86% of Mallard ducks were positive in the three provinces, respectively (Table 1). The positivity rate was not significant among the various species of ducks (*P* > 0.05). Among the positive samples, 64 (21.92%) of 292, 162 (41.65%) of 389 and 87 (52.1%) of 167 ducks were in the age ranges of 0 to 4, 4 to 8 and more than 8 weeks, respectively (Table 2). The DuCV prevalence in ducks more than 8 weeks old was 2.4- and 1.25-times greater than that in ducks 0 to 4 and 4 to 8 weeks old, respectively. The positivity rates were 23.23, 40.12 and 53.03% in Mulard ducks; 17.74, 42.11 and 51.16% in Cherry Valley Pekin ducks; and 22.58, 45.12 and 53.33% in Muscovy ducks aged 0 to 4 weeks, 4 to 8 weeks and more than 8 weeks, respectively. The positivity rates were 23.19 and 41.12% in Mallard ducks aged 0 to 4 weeks and 4 to 8 weeks; positivity in ducks aged more than 8 week was not recorded in this study (Table 2). The results prompted us to determine that ducks more than 4 weeks old were more susceptible to DuCV than those less than 4 weeks old in Mulard ducks, Cherry Valley Pekin ducks and Muscovy ducks (*P* < 0.05).

**Table 1:** Details of the bursa samples from different regions in southern and southwestern China

Farms	Province	Breed	Age (weeks)	Number tested	Number of positive ducks	Positive number (rate %) of DuCV	Number of Species ducks positive for DuCV/(%)	Number of ducks positive for DuCV/(%)
1	Guangdong	Mulard duck	2	24	5	20.83%	45/(39.47%)	86/(34.4%)
2	Guangdong	Mulard duck	6	13	5	38.46%		
3	Guangdong	Mulard duck	9	34	18	52.94%		
4	Guangdong,	Mulard duck	6	23	9	39.13%		
5	Guangdong	Mulard duck	6	20	8	40.00%		
6	Guangdong	Cherry Valley pekin duck	2	22	4	18.18%	24/(30%)	
7	Guangdong	Cherry Valley pekin duck	4	20	4	20.00%		
8	Guangdong	Cherry Valley pekin duck	7	38	16	42.11%		
9	Guangdong	Muscovy duck	9	15	8	53.33%	11/(40.74%)	
10	Guangdong	Muscovy duck	4	12	3	25.00%		
11	Guangdong,	Mallard duck	3	10	2	20.00%	6/(20.69%)	
12	Guangdong,	Mallard duck	4	19	4	21.05%		
13	Guangxi	Mulard duck	2	42	10	23.81%	40/(31.75%)	121/(34.38%)
14	Guangxi	Mulard duck	5	30	11	36.67%		
15	Guangxi	Mulard duck	6	36	15	41.67%		
16	Guangxi	Mulard duck	4	18	4	22.22%		
17	Guangxi	Cherry Valley pekin duck	9	35	17	48.57%	33/(41.77%)	
18	Guangxi	Cherry Valley pekin duck	10	24	13	54.17%		
19	Guangxi	Cherry Valley pekin duck	2	20	3	15.00%		
20	Guangxi	Muscovy duck	4	19	4	21.05%	15/(30.61%)	
21	Guangxi	Muscovy duck	5	30	11	36.67%		
22	Guangxi	Mallard duck	4	25	7	28.00%	33/(33.67%)	
23	Guangxi	Mallard duck	5	30	12	40.00%		
24	Guangxi	Mallard duck	5	28	11	39.29%		
25	Guangxi	Mallard duck	3	15	3	20.00%		
26	Yunnan	Mulard duck	7	22	9	40.91%	38/(43.68%)	106/(43.09%)
27	Yunnan	Mulard duck	7	18	8	44.44%		
28	Yunnan	Mulard duck	4	15	4	26.67%		
29	Yunnan	Mulard duck	9	32	17	53.13%		
30	Yunnan	Cherry Valley pekin duck	9	27	14	51.85%	14/(51.85%)	
31	Yunnan	Muscovy duck	6	22	10	45.45%	33/(39.76%)	
32	Yunnan	Muscovy duck	8	30	16	53.33%		
33	Yunnan	Muscovy duck	3	31	7	22.58%		
34	Yunnan	Mallard duck	6	27	12	44.44%	21/(42.86%)	
35	Yunnan	Mallard duck	5	22	9	40.91%		
Total	Three province	ducks		848	313			313/(36.91%)

**Table 2:** Age and species distribution of DuCV positive ducks detected in this study

species	Mulard duck			Cherry Valley pekin duck			Muscovy duck			Mallard duck			All ducks		
	Samples in all	positive	Positive rate (%)	Samples in all	positive	Positive rate (%)	Samples in all	positive	Positive rate (%)	Samples in all	positive	Positive rate (%)	Samples in all	positive	Positive rate (%)
0 < week≤4	99	23	23.23	62	11	17.74	62	14	22.58	69	16	23.19	292	64	21.92
4 < week≤8	162	65	40.12	38	16	42.11	82	37	45.12	107	44	41.12	389	162	41.65
Week> 8	66	35	53.03	86	44	51.16	15	8	53.33	NT^a^	NT^a^	NT^a^	167	87	52.10
Total	327	123	37.61	186	71	38.17	159	59	37.11	176	60	34.09	848	313	36.91

^a^not tested

### Multiple alignment and recombination analysis of DuCV

Sequence analyses showed that the full genomic lengths of the 19 DuCVs were 1987–1995 nt; the sequences were deposited into GenBank under the accession numbers MK814571-MK814589. Notably, the lengths of the novel DuCV strains GD150501 (MK814571), GD150502 (MK814572), GD180503 (MK814573), GD180504 (MK814574), GD190401 (MK814575), GD190402 (MK814576) and GD190403 (MK814577) (1987 bp) were due to a deletion mutation at the 966 site and were different from that of WS-GD01 (1988 bp) from Guangdong. Interestingly, the GX150511 (MK814578), GX190512 (MK814579), GX190509 (MK814580) and GX190510 (MK814581) strains were 1993 bp; the GX190511 (MK814582) and GX190512 (MK814583) strains were both 1995 bp, and there were also differences in the GX11049 (1988 bp) and LZ/11/0 (1994 bp) strains from Guangxi. Moreover, the sequence lengths of the YN180505 (MK814584) and YN180506 (MK814585;1988 bp) as well as the YN190410 (MK814586), YN190411 (MK814587), YN190412 (MK814588) and YN190415 (MK814589) (1987 bp) sequences were most similar to those of the YN24–2013 (1988 bp) and YN27–2013 (1987 bp) sequences from Yunnan. Multiple sequence alignment of the complete nucleotide sequences of the 19 novel DuCVs showed 81.7–99.3% homology with the other genome sequences in GenBank and 86.4–99.8% and 77.5–99.5% homology with the other ORF 1 and ORF 2 gene sequences in GenBank, respectively. Based on the whole-genome nucleotide sequences analysis, the DuCVs showed multiple mutations compared with Germany and Taiwan lineage strains (data not shown). The sequences MK814571-MK814577, MK814584 and MK814585 shared 6 major variable regions (residues 3 to 15, 31 to 64, 104 to 124, 143 to 159, 177 to 213, and 232 to 238) in the cap protein in DuCV-2, consistent with a previous report [6]. The DuCV sequences MK814586-MK814589 had identical substitutions in the DuCV-1 cap protein at positions 9 A/P, 82 R/H, 106 S/T, 135 K/E, 236 N/D (online Technical Appendix Figure S1 A). In addition, MK814588 and MK814589 also had identical substitutions at 28 L/V and 216 D/G in the rep protein. MK814571-MK814577 had identical substitutions in the DuCV-2 rep protein at position 22 E/D (online Technical Appendix Figure S1 B).

We analyzed 19 full-length sequences, and a putative recombination sites (at nt 989 and 1122) was detected in the genome of recombinant 3 strains (YN190411-China-Yunnan, YN190412-China-Yunnan, and YN190415-China-Yunnan) originating from the strains GD150501-China-Guangdong and GX190512-China-Guangxi.

### Phylogenetic analysis of DuCVs

**Fig. 1:**
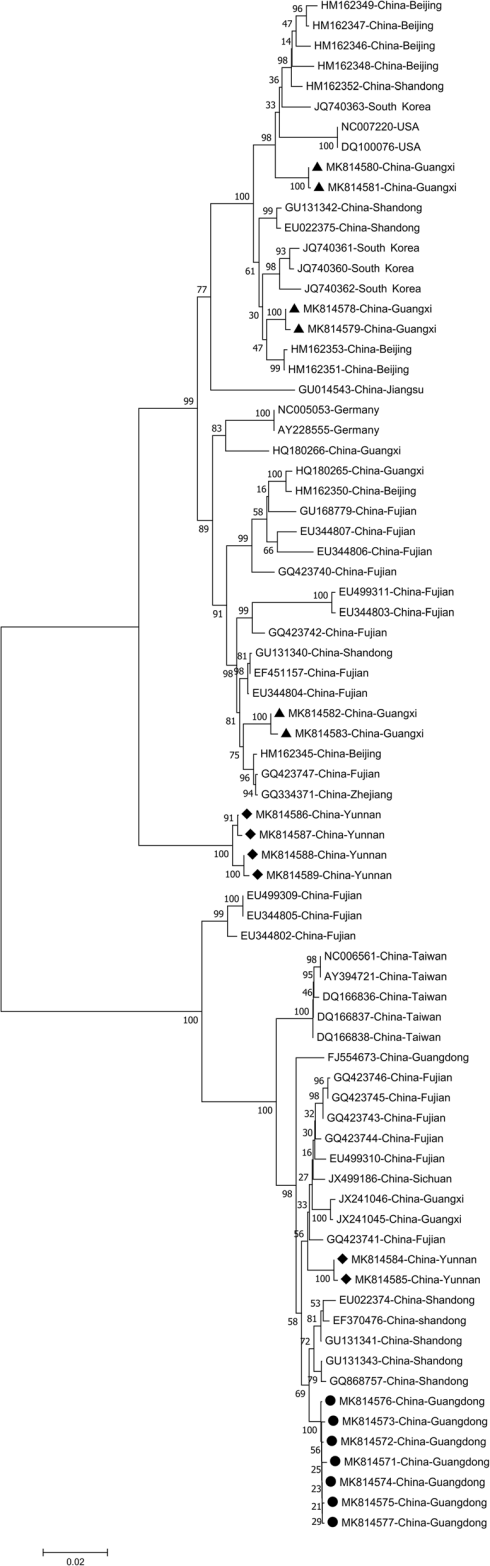
Phylogenetic analysis of novel duck circovirus (DuCV) whole-genome sequences using the neighbor-joining (NJ) method with 1000 bootstrap replications (Mega 7.0). DuCV-1-duck circovirus genotype 1, DuCV-2-duck circovirus genotype 2. The sequence of the circovirus detected in this study is marked with a symbol. DuCV strains from Guangdong are indicated with circles, those from Guangxi with a triangle, and those from Yunnan with a square. The other sequences were obtained from GenBank, and the accession numbers of these sequences are included in the phylogenetic tree

Phylogenetic analyses of the full-length genomes of the 19 novel sequences and 57 reference sequences obtained from GenBank indicated that the DuCVs could be divided into two distinct genetic groups: DuCV-1 (Group 1: Germany lineage) and DuCV-2 (Group 2: Taiwan lineage) (Fig. 1). The DuCVs from Guangdong (MK814571-MK814577) formed a distinct clade within DuCV-2, while the DuCVs from Yunnan (MK814584 and MK814585) clustered in one branch. The DuCVs from Guangxi (MK814586-MK814589) and Yunnan (MK814578-MK814583) clustered in another branch within DuCV-1. The results of the phylogenetic analysis indicated that two genetic groups of DuCVs had caused epidemics in ducks in three provinces.

## Discussion

As stated in previous reports, DuCV may be an immunosuppressive virus that may increase the pathogenicity of coinfecting agents. The classic symptoms are generally considered to be feather disorders, poor body condition and low weight for age. The virus can persist in the thymus, liver, spleen, kidney and BF, and among these organs, the bursa is the most predominant location for circovirus replication [5, 11, 13, 15, 16, 22, 23]. There are no existing vaccines or drugs to cure or prevent DuCV, resulting in serious economic and production losses in the poultry industry. The prevalence of DuCV has been extensively reported in multiple species of ducks and from many provinces and cities in China [20, 24].

In this study, we aimed to elucidate the epidemiology of DuCVs circulating in southern and southwestern China. PCR methods were used in a molecular epidemiological investigation of DuCV in the BF of dead ducks, and the results confirmed a high overall DuCV infection rate in four species of ducks from Guangdong, Guangxi and Yunnan Province. However, the positivity rates of DuCV by species and province were not significantly different. These results were similar to previous reports in which DuCV infection was ubiquitous, showing no species dependency [15, 18]. In addition, the positivity rates showed that Mulard ducks, Cherry Valley Pekin ducks and Muscovy ducks more than 4 weeks old were more susceptible than ducks less than 4 weeks old, consistent with a previous report [15].

Similar to those of other members of the DuCV family, the genomic sequence of the 19 DuCVs contained three major ORFs: ORF 1, ORF 2 and ORF 3 [22, 25]. According to the phylogenetic analyses of the complete genomes, the DuCV in this study belonged to two genotypes [6]. Compared to previous reports, the 19 DuCVs detected in this study belonged to the Group 1 Germany lineage or Group 2 Taiwan lineage [15]. Based on these results, we confirmed that among these 19 DuCVs, Group 1 was prevalent in Guangdong, Group 2 was prevalent in Guangxi and both groups were prevalent in Yunnan province. Interestingly, there were differences between the DuCVs identified in this study and other DuCVs from previous reports, and the genome lengths of the novel sequences GD150501, GD150502 GD180503, GD180504, GD190401, GD190402 and GD190403 were all 1987 nt, which is the shortest observed DuCV genome on record [15, 20, 24, 26]. When the sequences were compared with the other genomes in GenBank, it became clear that there was a deletion at the 966 site in the intragenic region in 7 DuCVs from Guangdong, and other DuCVs also showed multiple mutations in the complete sequences. This suggests that there are mutations in the novel DuCV gene in the poultry of southern and southwestern China. In addition, we found that potential recombination events occurred in 3 strains from Yunnan, originating in strains from Guangdong and Guangxi. Geographically, Guangxi, Guangdong, and Yunnan are adjacent to each other, and the DuCV sequences were similar, leading to recombination events occurring in Yunnan. Our hypothesis is that virus transmission may have occurred due to trade within the province. A limitation of this study was that we collected only dead ducks; and novel DuCV should be collected and detected in Mallard ducks in the future. Additionally, other virus genotypes may infect ducks but produce no clinical symptoms. Therefore, we cannot rule out the existence of other genotypes in this region, but whether they cause clinical symptoms requires further investigation.

## Conclusion

This study will help elucidate the epidemiological and molecular characteristics of DuCV in southern and southwestern China. The acquired data suggest that DuCVs are highly prevalent and that there were two distinct genetic groups of novel DuCV in 4 species of ducks in 2018 and 2019. The novel DuCVs can infect different breeds of ducks, and ducks over 4 weeks old were the most susceptible. We were the first to report variations in the complete genome lengths of and recombination in a novel DuCV in southern and southwestern China [15, 20, 24–26]. DuCVs in additional species of ducks remain to be detected, and no wild bird samples were collected. In the future, we need to investigate additional species in poultry and wild-birds to determine the exact origin and analyze the transmission and pathogenesis of DuCVs to aid in the development of more effective vaccines against various DuCVs.

## Methods

### Sample collection

A total of 848 BF samples were collected from four species of ducks ranging from 2 to 10 weeks in age that presented clinical symptoms (feathering disorders, growth retardation and general sickness) and later died. Upon death, BF extraction was conducted according to standard care guidelines. The BFs from 327 Mulard ducks, 186 Cherry Valley Pekin ducks, 159 Muscovy ducks, and 176 Mallard ducks from 53 duck farms in Guangdong (20), Yunnan (17) and Guangxi (16) provinces in China from 2018 to 2019 were removed and sectioned (Table 1).

### PCR detection of DuCV

Total viral DNA was extracted from 0.1 g of BF samples by a previously described method [24]. The DNA was used to detect DuCV pathogens by PCR using the common DuCV detection primers F1: (5′-ATATTATTACCGGCGC (C/T) TG-3′) and R1: (TCAGGAATCCCTG (A/C) AGGTGA-3′) [12]. The complete genome was amplified using the primers F2: (5′-TCCGGATCCGAAAAATCCAAATAC-3′) and R2: (5′-CCCGGATCCGGAACTGGACCAAC-3′) [17]. The specific PCR products were purified using an agarose gel DNA purification kit and cloned using a pMD18-T kit (Takara, Dalian, China). The positive recombinant clones from three independent amplifications of every strain were Sanger sequenced in both directions (Sangon Biotech, Shanghai, China).

### Statistical analysis

All one-sample t tests were performed using GraphPad Prism 6 (GraphPad Software, Inc.), and two-tailed *P* < 0.05 was defined as statistically significant.

### Sequence analysis

Genetic variation and phylogenetic relationships with the complete genomes from 76 DuCV sequences available in GenBank were analyzed (online Technical Appendix Table S1). Nucleotide sequence alignments and homology comparisons were made using the Clustal W method in the MegAlign program (DNASTAR, Madison, USA). The neighbor-joining (NJ) method was used to generate phylogenetic trees based on the aligned nucleotide sequences in MEGA v7.0 [4, 27]. To detect putative recombination breakpoints in the DuCV genome and to identify sequences that possibly originated from a recombination event, the RDP 4.24 program was used [28].

## Supplementary information

**Additional file 1: Figure S1.** Deduced amino acid sequence comparisons of the 19 DuCV strains in Cap (A) and Rep (B) protein. **Table S1.** Details of DuCV isolates used in this study and other isolates available in GenBank.

## Data Availability

The datasets used and/or analyzed during the current study are available from the corresponding author upon reasonable request.

## Abbreviations

BF: Bursa of Fabricius
Cap: Capsid protein
DuCV: Duck circovirus
DuCV-1: Duck circovirus genotype 1
DuCV-2: Duck circovirus genotype 2
NJ: Neighbor-joining
ORF: Open reading frame
Rep: Replication protein
