# Supplementary material for: Molecular survey of duck circovirus infection in poultry in southern and southwestern China during 2018 and 2019
Source: BMC Vet Res. 2020 Mar 5;16:80. doi: 10.1186/s12917-020-02301-x (PMC7059369; doi:10.1186/s12917-020-02301-x)
Supplement: Supplementary file 1 — Additional file 1: Figure S1. Deduced amino acid sequence comparisons of the 19 DuCV strains in Cap (A) and Rep (B) protein. Table S1. Details of DuCV isolates used in this study and other isolates available in GenBank. [file 12917_2020_2301_MOESM1_ESM.docx]

**Figure S1**

Deduced amino acid sequence comparisons of the 19 DuCV strains in Cap (A) and Rep (B) protein.


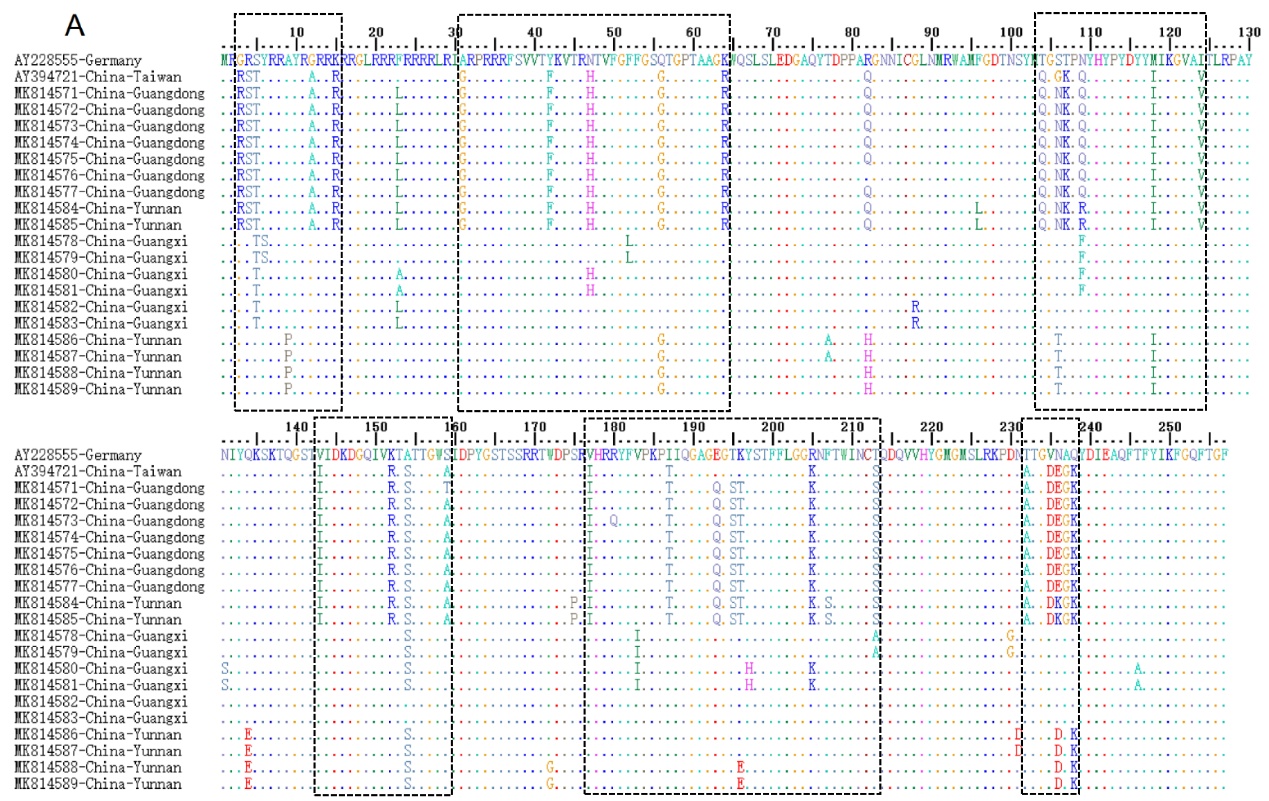


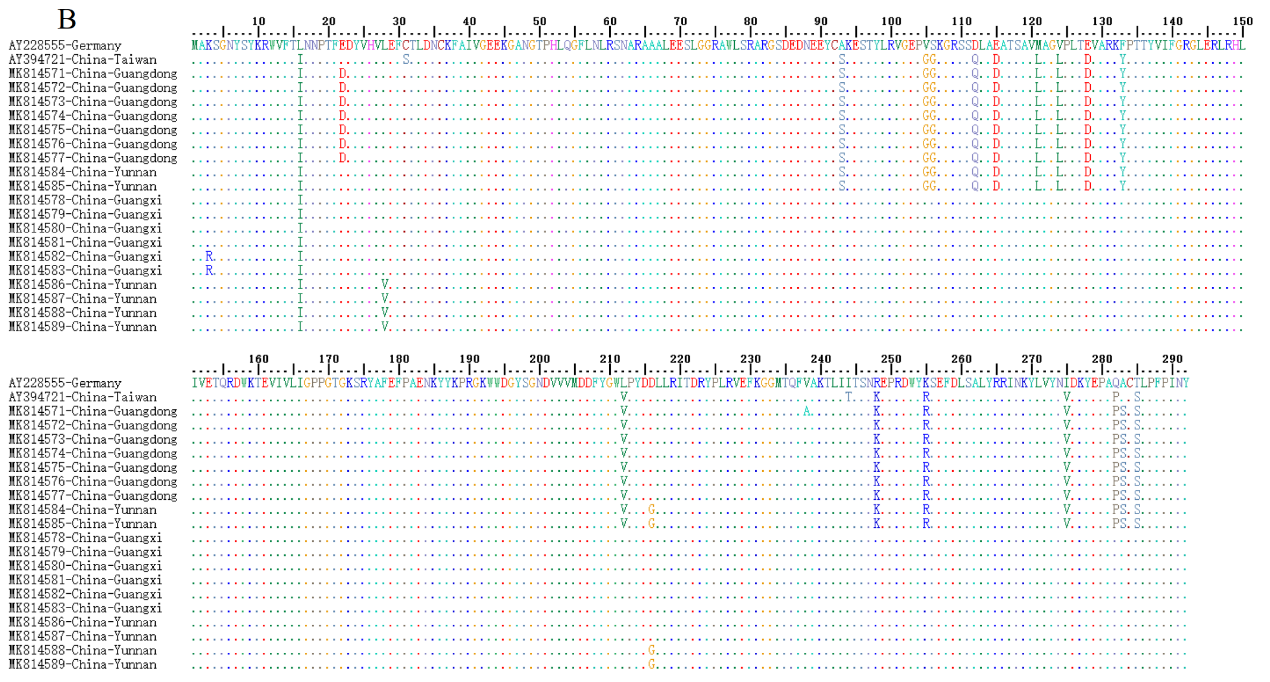


Note: The rectangles indicate major variable regions.

**Table S1.** Details of DuCV isolates used in this study and other isolates available in GenBank

| No. of isolates | Accession number | | Genome size (nt) | Collected time | Geographic Origin | Host |
| --- | --- | --- | --- | --- | --- | --- |
| 1 | AY228555 | 1996 | | 2003 | Germany | Mulard duck |
| 2 | AY394721 | 1988 | | 2002 | Taiwan, China | Muscovy duck |
| 3 | DQ100076 | 1991 | | 2005 | USA | Cherry Valley pekin duck |
| 4 | DQ166836 | 1988 | | 2002 | Taiwan, China | Muscovy duck |
| 5 | DQ166837 | 1988 | | 2005 | Taiwan, China | Muscovy duck |
| 6 | DQ166838 | 1988 | | 2005 | Taiwan, China | Muscovy duck |
| 7 | EF370476 | 1988 | | 2006 | Fujian, China | Muscovy duck |
| 8 | EF451157 | 1995 | | 2007 | Fujian, China | Muscovy duck |
| 9 | EU022374 | 1988 | | 2007 | Fujian, China | duck |
| 10 | EU022375 | 1991 | | 2007 | Shandong, China | duck |
| 11 | EU344802 | 1989 | | 2007 | Fujian, China | Muscovy duck |
| 12 | EU344803 | 1995 | | 2007 | Fujian, China | Mule duck |
| 13 | EU344804 | 1995 | | 2007 | Fujian, China | Mule duck |
| 14 | EU344805 | 1988 | | 2007 | Fujian, China | Muscovy duck |
| 15 | EU344806 | 1992 | | 2007 | Fujian, China | Cherry Valley pekin duck |
| 16 | EU344807 | 1992 | | 2007 | Fujian, China | Muscovy duck |
| 17 | EU499309 | 1988 | | 2007 | Fujian, China | Mule duck |
| 18 | EU499310 | 1988 | | 2008 | Fujian, China | Muscovy duck |
| 19 | EU499311 | 1995 | | 2008 | Fujian, China | Muscovy duck |
| 20 | FJ554673 | 1988 | | 2008 | Guangdong, China | Muscovy duck |
| 21 | GQ334371 | 1995 | | 2007 | Zhejiang, China | Muscovy duck |
| 22 | GQ423740 | 1995 | | 2008 | Fujian, China | Muscovy duck |
| 23 | GQ423741 | 1988 | | 2007 | Fujian, China | duck |
| 24 | GQ423742 | 1995 | | 2007 | Fujian, China | Muscovy duck |
| 25 | GQ423743 | 1988 | | 2009 | Fujian, China | Muscovy duck |
| 26 | GQ423744 | 1988 | | 2009 | Fujian, China | Muscovy duck |
| 27 | GQ423745 | 1988 | | 2009 | Fujian, China | Muscovy duck |
| 28 | GQ423746 | 1988 | | 2009 | Fujian, China | Muscovy duck |
| 29 | GQ423747 | 1995 | | 2008 | Fujian, China | duck |
| 30 | GQ868757 | 1988 | | 2006 | Fujian, China | Muscovy duck |
| 31 | GU014543 | 1995 | | 2009 | Jiangsu, China | duck |
| 32 | GU131340 | 1995 | | 2008 | Shandong, China | Cherry Valley pekin duck |
| 33 | GU131341 | 1988 | | 2008 | Shandong, China | Cherry Valley pekin duck |
| 34 | GU131342 | 1991 | | 2008 | Shandong, China | Cherry Valley pekin duck |
| 35 | GU131343 | 1988 | | 2008 | Shandong, China | Cherry Valley pekin duck |
| 36 | GU168779 | 1995 | | 2008 | Fujian, China | Muscovy duck |
| 37 | HM162345 | 1995 | | 2009 | Beijing, China | Cherry Valley pekin duck |
| 38 | HM162346 | 1993 | | 2008 | Beijing, China | Cherry Valley pekin duck |
| 39 | HM162347 | 1994 | | 2008 | Beijing, China | Cherry Valley pekin duck |
| 40 | HM162348 | 1993 | | 2008 | Beijing, China | Cherry Valley pekin duck |
| 41 | HM162349 | 1994 | | 2008 | Beijing, China | Cherry Valley pekin duck |
| 42 | HM162350 | 1994 | | 2010 | Beijing, China | Cherry Valley pekin duck |
| 43 | HM162351 | 1996 | | 2010 | Beijing, China | Cherry Valley pekin duck |
| 44 | HM162352 | 1993 | | 2010 | Shandong, China | Cherry Valley pekin duck |
| 45 | HM162353 | 1996 | | 2010 | Beijing, China | Cherry Valley pekin duck |
| 46 | HQ180265 | 1994 | | 2008 | Guangxi, China | Muscovy duck |
| 47 | HQ180266 | 1994 | | 2009 | Guangxi, China | Mulard duck |
| 48 | JQ740360® | 1993 | | 2011 | South Korea | duck |
| 49 | JQ740361 | 1993 | | 2011 | South Korea | duck |
| 50 | JQ740362 | 1993 | | 2011 | South Korea | duck |
| 51 | JQ740363 | 1993 | | 2011 | South Korea | duck |
| 52 | JX241045 | 1988 | | 2011 | Guangxi, China | duck |
| 53 | JX241046 | 1988 | | 2011 | Guangxi, China | duck |
| 54 | JX499186 | 1988 | | 2012 | Sichuan, China | Muscovy duck |
| 55 | NC005053 | 1996 | | 2003 | Germany | Mulard duck |
| 56 | NC006561 | 1988 | | 2002 | Taiwan, China | Muscovy duck |
| 57 | NC007220 | 1991 | | 2005 | USA | Cherry Valley pekin duck |
| 58 | MK814571 | 1987 | | 2018 | Guangdong, China | Mulard duck |
| 59 | MK814572 | 1987 | | 2018 | Guangdong, China | Muscovy duck |
| 60 | MK814573 | 1987 | | 2018 | Guangdong, China | Mallard duck |
| 61 | MK814574 | 1987 | | 2018 | Guangdong, China | Cherry Valley pekin duck |
| 62 | MK814575 | 1987 | | 2019 | Guangdong, China | Cherry Valley pekin duck |
| 63 | MK814576 | 1987 | | 2019 | Guangdong, China | Mallard duck |
| 64 | MK814577 | 1987 | | 2019 | Guangdong, China | Cherry Valley pekin duck |
| 65 | MK814578 | 1993 | | 2018 | Guangxi, China | Mulard duck |
| 66 | MK814579 | 1993 | | 2018 | Guangxi, China | Muscovy duck |
| 67 | MK814580 | 1993 | | 2019 | Guangxi, China | Mallard duck |
| 68 | MK814581 | 1993 | | 2019 | Guangxi, China | Cherry Valley pekin duck |
| 69 | MK814582 | 1995 | | 2019 | Guangxi, China | Cherry Valley pekin duck |
| 70 | MK814583 | 1995 | | 2019 | Guangxi, China | Muscovy duck |
| 71 | MK814584 | 1988 | | 2018 | Yunnan, China | Muscovy duck |
| 72 | MK814585 | 1988 | | 2018 | Yunnan, China | Cherry Valley pekin duck |
| 73 | MK814586 | 1987 | | 2019 | Yunnan, China | Muscovy duck |
| 74 | MK814587 | 1987 | | 2019 | Yunnan, China | Mulard duck |
| 75 | MK814588 | 1987 | | 2019 | Yunnan, China | Muscovy duck |
| 76 | MK814589 | 1987 | | 2019 | Yunnan, China | Mallard duck duck |
